# GC Gene Polymorphism and Unbound Serum Retinol-Binding Protein 4 Are Related to the Risk of Insulin Resistance in Patients With Chronic Hepatitis C: A Prospective Cross-Sectional Study

**DOI:** 10.1097/MD.0000000000003019

**Published:** 2016-03-11

**Authors:** Beatriz Mateos-Muñoz, Elena García-Martín, María J. Torrejón, María J. Devesa-Medina, Gara Esguevillas, María C. Cárdenas, Cristina Fernández, Miguel Carballo, José A. Agúndez, José M. Ladero

**Affiliations:** From the Services of Gastroenterology (BM-M, MJD-M, JML), Clinical Laboratory (MJT, MCC), and Clinical Epidemiology (CE), Hospital Clínico San Carlos, Instituto de Investigación Sanitaria del Hospital Clínico San Carlos (IdISSC), Madrid, Spain (CF), Department of Pharmacology, Universidad de Extremadura (EG-M, GEN, JAA), Laboratory of Molecular Genetics, Hospital de Terrassa, Terrassa, Barcelona, Spain (MC), and Department of Medicine, Universidad Complutense, Madrid, Spain (MJD-M, JML).

## Abstract

Insulin resistance (IR) is found in chronic hepatitis C (CHC) more frequently than in other chronic liver diseases.

Prospective cross-sectional study to evaluate a wide multitest panel to identify factors related with IR in CHC and their possible interactions.

In 76 patients with CHC we performed a series of routine laboratory analysis as well as specifically designed serum biochemical tests [retinol, retinol-binding protein 4 (RBP4), 25-OH vitamin D, Vitamin E, lipopolysaccharide-binding protein (LBP), interleukin-6 (IL-6), and cystatin C]. The single nucleotide polymorphisms rs7041 and rs4588 *GC-DBP* (group-specific component-Vitamin D-binding protein), rs738409 *PNPLA3* (patatin-like phospholipase domain containing 3), and rs12979860 *IL28B* (interleukin-28 B) genes were determined. Insulin sensitivity was established with the HOMA-IR and IR was diagnosed when HOMA-IR > 3. Fibrosis staging was assessed with liver biopsy or transient elastography.

After backward logistic regression analysis, independent variables associated with IR were Gc1s/Gc1s DBP phenotype, that results from the homozygous carriage of the rs7041G/rs4588C haplotype (*P* = 0.033); low retinol/RBP4 ratio, reflecting a greater rate of unbound RBP4 (*P* = 0.005); older age (*P* = 0.01); high serum tryglicerides (*P* = 0.026); and advanced (F3–F4) fibrosis stage. The AUROC provided by the multivariate model was 0.950 (95% CI = 0.906–0.993).

In addition to previously known ones, the Gc1s/Gc1s phenotype variant of DBP and the unbound fraction of plasma RBP4 may be considered as factors related with the incidence, and possibly the risk, of IR in CHC patients.

## Abstract 2

Supplemental Digital Content is available in the text

## INTRODUCTION

Insulin resistance (IR) is more frequent in chronic hepatitis C (CHC) than in other chronic liver diseases.^1^ Hepatitis C virus (HCV) chronic infection directly causes IR by interfering with insulin signaling cascade,^2^ mainly through the interaction between the HCV core protein at several steps of this cascade,^3,4^ but also by upregulating the production of proinflammatory cytokines, such as tumor necrosis factor α (TNF-α) and interleukin-6 (IL-6). IR in CHC has been related with the progression of liver fibrosis in CHC,^1^ with a lower rate of success of interferon-based therapy,^5^ and with a greater risk of developing hepatocellular carcinoma (HCC).^6^ IR is the key factor for developing the so called “metabolic syndrome” (MS). MS is characterized by a constellation of cardiovascular risk-factors and morbidities that include the presence of at least three of the following components: elevated fasting glucose, elevated triglycerides, elevated blood pressure, elevated waist circumference, and low high-density lipoprotein (HDL) cholesterol.^7,8^ MS is associated with a chronic low-grade inflammatory estate.^9^ Nonalcoholic fatty liver disease (NAFLD) is considered as the hepatic manifestation of the metabolic syndrome.^10^ Therefore, it is easy to hypothesize that IR, NAFLD, derangements of serum lipid profile and a wide range of nonspecific inflammatory markers (ie, reactive C protein or IL-6) are interdependent phenomena although it may be difficult to establish which is (or are) the primary pathogenic event(s) for IR and which others are consequences or simply biochemical markers of IR, MS, and NAFLD.

Several GWAS in patients with CHC have identified a wide range of single nucleotide polymorphisms (SNPs) which have been located to genes related to many aspects of the natural history of chronic HCV infection.^11^ Among them, the *IL28B* rs12979860 CT polymorphism has been shown to strongly influence the rate of both spontaneous and IFN-induced viral clearance, probably by inducing differences in the baseline expression of IFN-activated genes.^12^ A strong association between rs738409 CG SNP at the *PNPLA3* (patatin-like phospholipase domain containing 3) and steatosis of the liver was originally described in patients with NAFLD^13^ and its more severe form (NASH—nonalcoholic steatohepatitis),^14^ but it has been also found in patients with CHC.^15^

Vitamin D exerts immunomodulatory effects in CHC.^16^ The synthesis, transport, and physiological effects of Vitamin D depend on the sequential function of several enzymatic pathways that are coded by highly polymorphic genes.^17^ In a previous study we analyzed the influence of polymorphisms at *CYP27B1* gene—that regulates the renal 1-hydroxylation of 25-OH-Vitamin D)—and *VDR* gene—that codes for the vitamin D transmembrane receptor—on the response to IFN-based therapy.^18^ Vitamin D-binding protein (DBP), also known as group-specific component protein (Gc) is the major serum transporter protein for Vitamin D.^19^ The *Gc* or *VDR* gene is polymorphic at 2 codon in exon 11 which give rise to 3 variants of the gene product, called respectively Gc 1F, Gc 1s, and Gc 2.^20^ A possible association of this polymorphism with IR in otherwise healthy subjects^21^ and with gestational diabetes mellitus^22^ has been reported.

The aim of this study has been to explore the possible association of polymorphic traits at *Gc*, *PNPLA3*, and *IL28B* genes with IR in patients with CHC and to detect if any interaction exists among them and a wide range of metabolic, inflammatory, biochemical, and virological parameters.

## PATIENTS AND METHODS

This is a prospective cross-sectional study including chronically HCV-infected outpatients attending to our Liver Unit from September 2013 to May 2014. In these patients, visits are scheduled at a 6 months interval, and therefore, nearly all the possible candidates were reviewed during the inclusion period. Inclusion criteria were active chronic infection with HCV for more than 6 months; known METAVIR stage of liver fibrosis^23^ disclosed by liver histology or transient elastography (for Fibroscan^®^ staging we have used the cutoff points proposed by Castera et al^24^) within the previous 12 months, and written informed consent. Exclusion criteria were coinfection with hepatitis B and/or human immunodeficiency viruses; current drinking of >40 g/day of ethanol; any anti-HCV therapy in the previous 12 months; diabetes mellitus; estimated glomerular filtrate <60 mL/min/1.73 m^2^ and, decompensated cirrhosis (criteria of decompensation were current or past ascites, hepatic encephalopathy, bleeding varices, hepatocellular carcinoma, and total serum bilirubin >3.0 mg/dl. Ascites were excluded with ultrasonography performed within the previous month. All patients provided written informed consent according with the Declaration of Helsinki. The study was approved by the Ethics Committee of the Hospital Clínico San Carlos, Madrid, Spain.

For each patient, all the analytical studies were performed in the same day. A venous blood sample was collected after overnight fast using a Vacutainer system (Becton Dickinson®, Franklin Lakes, NJ). After 30 minutes, blood samples were centrifuged during 10 minutes in a refrigerated centrifuge and serum samples were stored at 4°C or at −80° C until analysis. Height and body weight were measured to estimate the body mass index (weight in kg/height in m^2^).

Routine hematological, biochemical, and virological analysis were performed by standard tests at our laboratories as described elsewhere.^25^ The whole relationship of performed determinations is shown in supplementary material (Table S1). Methods specifically performed for this study were as follows: serum retinol and tocopherol measurements were performed using a Vitamin A-E kit from Chromsystems Diagnostics® (Munich, Germany) on a Shidmazu HPLC with UV detection at 325 and 295 nm. The calibration standard is traceable to NIST 968e reference material. Total 25(OH) vitamin D determination was measured by a competitive direct immunoassay using chemoluminiscency on an Architect i1000 analyzer (Abbott Diagnostics, Wiesbaden, Germany). Retinol binding protein (RBP) and cystatin C were measured by immunonephelometry on a BN Prospec analyzer (Siemens Healthcare Diagnostics, Marburg, Germany). Serum creatinine was measured by means of the modified kinetic Jaffé method using a Beckman Coulter AU 5400 (Beckman Coulter, Brea, CA). Insulin levels were analyzed with an immunoassay IMMULITE 2000 Insulin (Siemens®) and the HOMA-IR (Homeostasis Model Assessment) was calculated according to the formula: 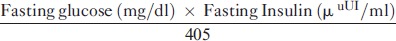


A HOMA-IR > 3 was considered as an indicator of IR, according with Moucari et al.^26^

LBP (lipopolysaccharide-binding protein) was measured in serum with a solid-phase 2-site chemiluminescent immunometric assay in an Immulite 1000 analyzer (Siemens Healthcare Diagnostics) and the IL6 was performed with an electrochemiluminescence immunoassay “ECLIA” in a Cobas E411® (Roche Diagnostics, Basel, Switzerland).

Four SNPs were studied by means of TaqMan probes (Thermo Fisher Scientific, Alcobendas, Madrid, Spain). These included the SNP rs12979860, corresponding to the *IL28B* gene, rs738409 corresponding to the *PNPLA3* gene, and 2 SNPs corresponding to the *Gc* gene, designated as rs7041 and rs4588. These SNPs were selected on the basis of their allele frequencies and clinical associations. Commercial primers were used for the detection of the SNPs (C___7820464_10, C___7241_10, C___3133594_30, and C___8278879_10, respectively; Thermo Fisher Scientific). The detection was carried out by qPCR in an Eppendorf Realplex thermocycler (Eppendorf, Madrid, Spain). The amplification conditions were as follows: After a denaturation time of 10 minutes at 96°C, 45 cycles of 92°C 15 seconds 60°C 90 seconds were carried out and fluorescence was measured at the end of every cycle and at endpoint. All samples were determined by triplicate and genotypes were assigned both, by the gene identification software (RealPlex 2.0, Eppendorf) and by analysis of the reference cycle number for each fluorescence curve, calculated by the use of CalQPlex algorithm (Eppendorf).

For technical validation purposes, the amplified fragments for 20 individuals carrying every possible genotype were sequenced, and in all cases the genotypes fully corresponded with those detected with fluorescent probes.

Haplotype reconstruction for the *GC* SNPs was performed using the program PHASE v2.1.1.^27^

We used the default model for recombination rate variation with 1000 iterations, 500 burn-in iterations and a thinning interval of 1 as described elsewhere.^28^

Fibrosis stage was established by liver biopsy in 30 patients and by transient elastography in the remaining 46 and was categorized in 4 (1–4) METAVIR categories, as transient elastography does not discriminate between F0 and F1. Liver steatosis was defined, but not graded, by the presence of fat in the liver biopsy, when available, or of a pattern suggestive of steatosis in ultrasonography in the remaining cases.

### Statistical Analysis

Continuous variables, expressed as median and interquartile (IQ) range, were compared with the Student’ *t* test or the Mann–Whitney *U* test, each when adequate, depending on their Gaussian distribution. Categorical variables, expressed as count and percentage, were compared with the χ^2^ or the Fisher exact tests, each when appropriate, and the effect of differences was established by calculating the odds ratio with the 95% confidence interval. A *P*-value < 0.05 or a confidence interval not including the unit were considered significant. A stepwise logistic regression analysis was performed to evaluate the independent factors associated with IR by analyzing the covariants with *P* < 0.05 in the univariate analysis. The statistical analysis was carried out with the SPSS software 22.0 (SPSS, Inc., Chicago, IL) and with EpiDat 3.1 software (Junta de Galicia, Spain) for specific tests.

## RESULTS

A total of 81 patients who satisfied all the requisites to be included were asked to participate in the study. One patient refused to provide a blood sample for genetic studies, in a second patient the serum samples for nonroutine biochemical analysis were lost and in 3 patients DNA amplification failed. The remaining 76 patients (36 male, median age 55 years, IQ range 49–63.5) were included in the study and their data were fully available for analysis. All patients were white Spaniards of Spanish European ancestry, mainly from Central and Southern peninsular Spain.

Table S1 (see Supplementary Material online) shows the median values with IQ range of all the variables included in the study in the whole group and the comparative analysis between patients without (HOMA-IR ≤ 3, n = 34) and with (HOMA-IR 3, n = 42) IR.^26^Table 1 is limited to those results showing significant differences in the univariate analysis. Sixty-five patients (85.5%) were infected with HCV genotype 1 (mostly 1b) and 5 (6.6%) with genotype 3. Viral load was classified as low (≤400,000 IU/mL) in 14 patients versus high (>400,000 IU/mL) in 62 patients, according to Witthoft et al.^29^

**TABLE 1:**
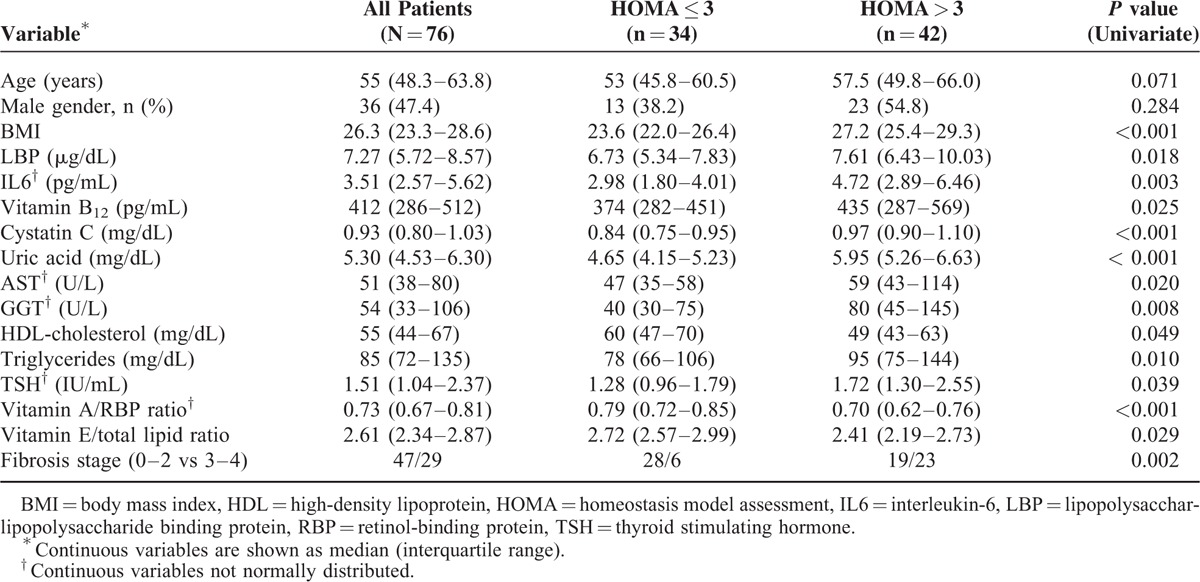
Significant Differences Related to Insulin Resistance Found at the Univariate Analysis of All Included Nongenetic Variables

The distribution of the studied genetic polymorphisms is shown in Table 2, as are comparisons between subgroups defined according to the IR status. In the univariate analysis, no differences were found in the distribution of the *IL28B* and *PNPLA3* SNPs. Both polymorphisms were in Hardy–Weinberg equilibrium and no linkage disequilibrium was found between them. The allele frequencies of the studied SNP at the *IL28B* gene were consistent with those previously reported among Spaniards.^30,31^

**TABLE 2:**
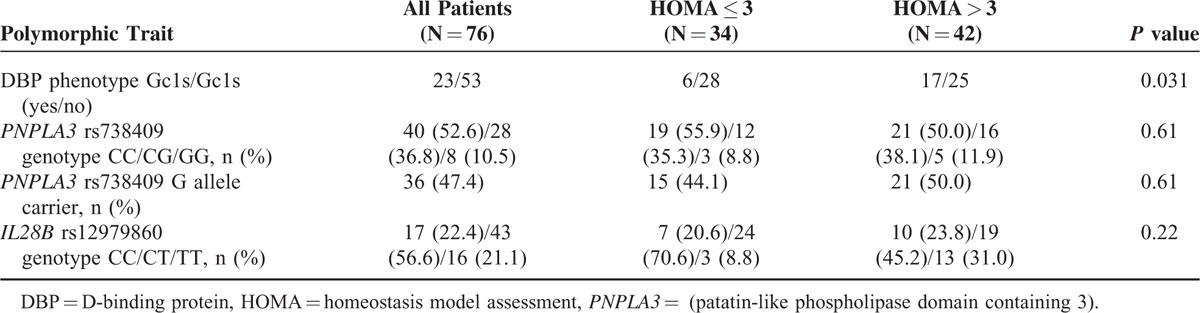
Genetic Polymorphisms Included in the Study

There was a significant excess of patients carrying the Gc1s/Gc1s DBP phenotype, that is defined by the homozygosity for the biallelic haplotype rs7041G/rs4588C at the exon 11 of the *Gc* (*DBP*) gene,^20^ in the group of patients with IR (*P* = 0.031). However, 25(OH) vitamin D serum levels were not related with the Gc phenotype (Gc1s/Gc1s = 23.8 ng/dl [IQR 17.9–31.4] vs non Gc1S/Gc1S = 21.8 ng/dl [IQR 14.4–30.1], *P* = 0.342).

The carrier state of the rs738409G allele of the *PNPLA3* gene was not related with presumptive inflammatory markers, both direct (LBP, IL-6, and serum cystatin C), and inverse (prealbumin and RBP4), nor with metabolic parameters related to the metabolic syndrome (blood lipids, uric acid, serum ferritin, TSH, and T4) (data not shown). The stage of fibrosis was established by liver biopsy in 30 patients and transient elastography in the remaining 46. There was a nonsignificant excess of more advanced stages of fibrosis (F3–F4) among carriers of the rs738409 G allele (*P* = 0.15) and all homozygous carriers of this allele had advanced fibrosis. The rate of roughly estimated steatosis of the liver was not related to the *PNPLA3* polymorphism (data not shown).

**FIGURE 1:**
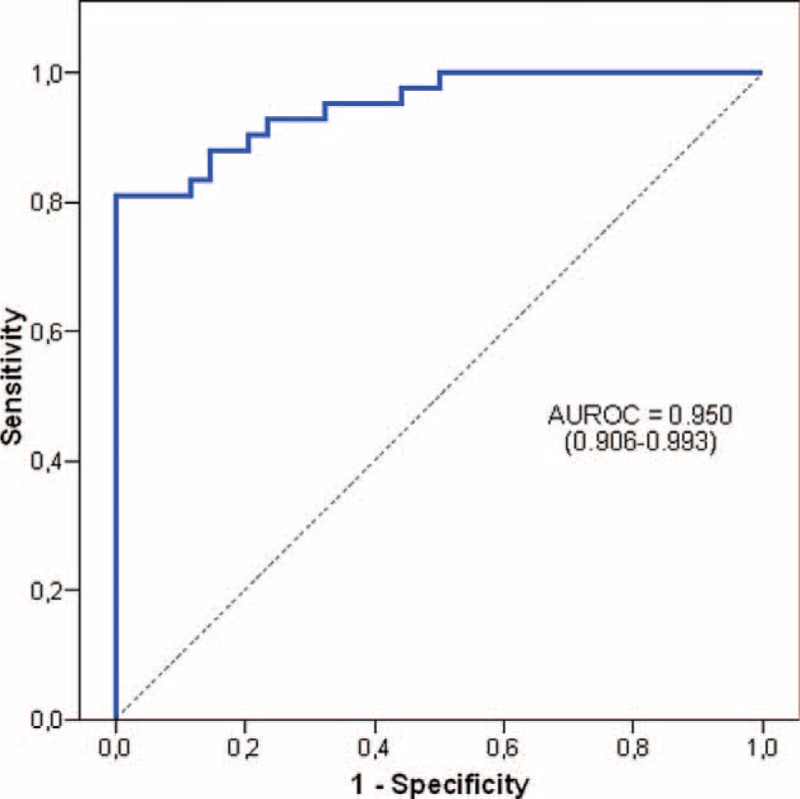
Receiver operating characteristic curve provided by the model constructed to establish the predictive value for insulin resistance (HOMA-IR > 3).

All the parameters related with IR in the univariate analyses shown in Tables 1 and 2 were included in the multivariate analysis. Only 5 variables (older age, higher triglyceride levels, lower vitamin A/RBP ratio, advanced fibrosis stage, and Gc1s/Gc1s phenotype) were significantly related to the risk of IR (Table 3). Figure 1 shows the receiver operating characteristic (ROC) curve provided by the model constructed to establish predictive values for IR (AUROC = 0.950, 95% CI: 0.906–0.993).

**TABLE 3:**
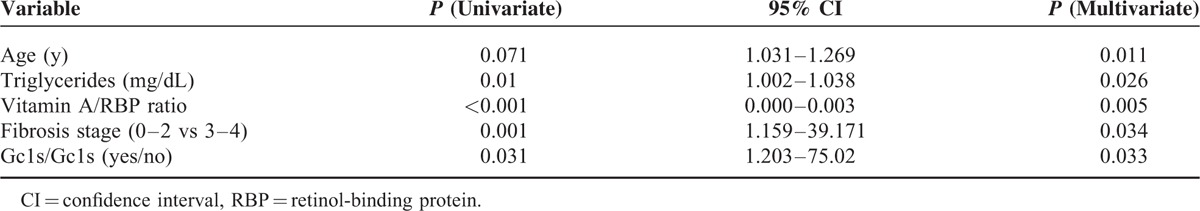
Variables Significantly Related to Insulin Resistance in the Multivariate Analysis

## DISCUSSION

Up to our knowledge this is the first study that shows an independent relation between a well-known polymorphic trait at the *Gc* gene, also known as the *DBP* gene, and the risk of IR in patients with CHC. Patients carrying the Gc1s/Gc1s protein phenotype had significantly higher values of HOMA-IR (*P* = 0.002) and—most important—a greater percentage of cases with defined insulin resistance (HOMA-IR > 3).

The association of IR with the *Gc* gene polymorphism has been studied in several ethnic groups, mostly of non-Caucasian origin. This is important because the distribution of this polymorphism shows a great interethnic variability and results are not comparable. Moreover, most reports did not include the analysis of the HOMA index, but only considered blood glucose or insulin levels, both fasting and after oral glucose tolerance test, separately,^32–34^ with the exception of Hirai et al^21^ in their study on 82 Japanese subjects with normal glucose tolerance; only 6 of these patients carried the Gc1s/Gc1s phenotype, but HOMA was significantly higher in them than in patients with Gc1f/Gc1f phenotype, used as reference and that was present only in 1 patient of our group.

Hepatocellular carcinoma (HCC) is a frequent complication of HCV-induced CHC in advanced, mainly cirrhotic, stage. Lange et al^35^ studied the rs22822679 SNP at the *Gc* gene in CHC patients with and without HCC and found an excess of the minor G allele, that has some influence on the serum levels of 25(OH) vitamin D and that is strong linkage disequilibrium with the rs7041 polymorphism included in our study.^36^ Unfortunately, these authors did not analyze aspects related with glucose metabolism in their control group of 4325 CHC patients without HCC.

A second interesting finding of our study is the significant relation between IR and low values of the retinol-to-RBP4 ratio. Retinol binding protein 4 transports 95% of serum retinol^37^ and both parameters are strongly correlated (r = 0.884 in our study). Hence, the serum level of retinol strictly depends on the hepatic synthesis and excretion of RBP4, that, in turn, decreases in parallel to increasing liver damage^38–40^ and inflammation.^41^ It has been suggested that a retinol-to-RBP4 ratio <0.8 reflects retinol deficiency better than serum retinol level.^42^ However, RBP4 has been recently identified as an adipokine and a relative excess of RBP4 in relation to retinol (ie, an increase of the free or unbound fraction of RBP4) may reflect IR instead of retinol deficiency,^43^ a possibility that is in accordance with our finding of higher levels of elevated unbound RBP4 that were independent of the vitamin A level and the fibrosis stage of CHC, but closely correlated with serum IL-6, a well-known inflammatory cytokine (rho = −0.515 in our study).

The remaining factors that we have found to be related with IR should be commented in brief. Increasing age is associated with the emergence of MS and IR, and not only with the natural, and highly variable, course of HCV chronic infection.^44^ A high plasma triglyceride level is one of the most conspicuous metabolic findings in MS,^8^ whereas high uric acid levels are frequently found in MS and linked to insulin sensitivity index, probably through its close relationship with triglyceride levels,^45^ thus explaining why uric acid it is in the limit of significance in the multivariate analysis and excluded from the predictive model for IR that we propose in this study. Our results also confirm that IR is associated with an accelerate progression of liver fibrosis in CHC.^1^ Two independent groups^46,47^ found lower values for HOMA-IR in nondiabetic CHC patients carrying the rs12979860 CC *IL28B* genotype than in carriers of the other two possible genotypes (CT and TT), but our results and those of Degasperi et al^48^ and Lemoine et al^49^ do not confirm this relationship.

Our study has several limitations. The most important is the small sample size, although more than 95% of adequate available patients were included. On the other side, the stage of fibrosis was established by 2 different methods (liver biopsy and transient elastography) not always coincidental. In addition, the lack of histological study in 40 cases impeded us to know the incidence and degree of liver steatosis to evaluate the suggested role of the *PNPLA3* polymorphism on the risk of developing this important aspect of the liver damage in CHC.^15^ In spite of these drawbacks, we can conclude that the novel findings provided by this study are that a common polymorphic trait at the *DBP4* gene, causing the expression of the Gc1s/Gc1s phenotype, and the possible overexpression of RBP4 (or alternatively, a relative deficiency of vitamin A) are linked to the risk of developing IR in patients with CHC.

## Supplementary Material

Supplemental Digital Content
