# Supplementary material for: GC Gene Polymorphism and Unbound Serum Retinol-Binding Protein 4 Are Related to the Risk of Insulin Resistance in Patients With Chronic Hepatitis C: A Prospective Cross-Sectional Study
Source: Medicine (Baltimore). 2016 Mar 11;95(10):e3019. doi: 10.1097/MD.0000000000003019 (PMC4998900; doi:10.1097/MD.0000000000003019)
Supplement: Supplemental Digital Content [file medi-95-e03019-s001.docx]

**Supplemental Table s1. Complete relation and intergroup comparations of performed tests***

| Parameter | All patients  (N = 76) | HOMA ≤ 3  (N = 34) | HOMA > 3  (N = 42) | *p* value |
| --- | --- | --- | --- | --- |
| Age (years) | 55 (49-63.5) | 53 (46-60) | 57.5 (49.2-65.5) | 0.071 |
| Male gender, n (%) | 36 (47.4) | 13 (38.2) | 23 (54.8) | 0.284 |
| BMI | 26.3 (23.3-(28.6) | 23.6 (22.0-26.4) | 27.2 (25.4-29.3) | < 0.001 |
| Hemoglobin (g/dL) | 14.9 (14.1-15.9) | 14.8 (14.1-15.4) | 15.1 (14.2-16.4) | 0.67 |
| Platelets (x 10^3^/μL) | 183 (138-224) | 183 (148-227) | 181 (120-221) | 0.78 |
| INR** | 0.9 (0.8-1.0) | 0.9 (0.8-0.9) | 0.9 (0.8-1.0) | 0.24 |
| Iron (µg/dL) | 123 (87-154) | 120 (87-147) | 129 (88-158) | 0.73 |
| Ferritin** (ng/dL) | 159 (83-281) | 133 (74-206) | 202 (91-349) | 0.06 |
| Folic acid **(ng/mL) | 8.2 (6.4-11.7) | 8.2 (6.4-11.1) | 8.4 (6.2-12.7) | 0.84 |
| Vitamin B12 (pg/mL) | 412 (286-512) | 374 (282-451) | 435 (287-569) | 0.025 |
| Total serum protein (g/dL) | 7.3 (7-7.6) | 7.1 (7.0-7.5) | 7.3 (7.1-7.6) | 0.34 |
| Albumin (g/dL) | 4.3 (4.0-4.5) | 4.3 (4.1-4.5) | 4.2 (3.9-4.5) | 0.061 |
| Fibrinogen (mg/dL) | 335 (275-384) | 341 (279-384) | 327 (274-384) | 0.43 |
| Glucose (mg/dL) | 97 (90-105) | 92 (87-97) | 102 (95-112) | < 0.001 |
| Creatinine^4^ (mg/dL) | 0.90 (0.81-1.0) | 0.87 (0.76-0.98) | 0.90 (0.81-1.0) | 0.32 |
| EGF (ml/min) | 81 (72-90) | 81 (76-94) | 81 (71-87) | 0.63 |
| Cystatin C (mg(dL) | 0.93 (0.80-1.03) | 0.84 (0.75-0.95) | 0.97 (0.90-1.10) | < 0.001 |
| Uric acid (mg/dL) | 5.30(4.53-6.30) | 4.65 (4.15-5.23) | 5.95 (5.26-6.63) | < 0.001 |
| LDH (U/L) | 392 (347-436) | 366 (344-426) | 394 (361-452) | 0.10 |
| ALT **(U/L) | 63 (45-95) | 57 (37-85) | 69 (53-104) | 0.06 |
| AST **(U/L) | 51 (38-80) | 47 (35-58) | 59 (43-114) | 0.020 |
| AST/ALT ratio | 0.82 (0.69-1.03) | 0.81 (0.64-1.03) | 0.85 (0.69-1.06) | 0.81 |
| GGT **(U/L) | 54 (33-106) | 40 (30-75) | 80(45-145) | 0.008 |
| Alkaline phosphatase (U/L) | 80 (64-105) | 75 (61-87) | 89 (66-114) | 0.09 |
| Total bilirubin (mg/dL) | 0.8 (0.6-1.2) | 0.7 (0.5-0.9) | 0.8 (0.6-1.2) | 0.063 |
| Cholesterol^4^ (mg/dL) | 176 (155-195) | 178 (156-207) | 168 (152-192) | 0.31 |
| HDL cholesterol (mg/dL) | 55 (44-67) | 60 (47-70) | 49 (43-63) | 0.049 |
| LDL cholesterol (mg/dL) | 97 (79-121) | 99 (80-130) | 93 (77-119) | 0.27 |
| Triglycerides** (mg/dL) | 85 (72-135) | 78 (66-106) | 95 (75-144) | 0.010 |
| Free thyroxin (pg/mL) | 8.6 (7.9-9.4) | 8.5 (8.0-9.3) | 8.7 (7.6-9.6) | 0.33 |
| TSH (IU/mL) | 1.51 (1.04-2.37) | 1.28 (0.96-1.79) | 1.72 (1.30-2.55) | 0.039 |
| Prealbumin (mg/dL) | 19.9 (16.4-24.1) | 19.9 (17.3-24.1) | 19.9 (15.4-24.1) | 0,41 |
| Vitamin A^4^ (mg/L) | 0.36 (0.27-0.42) | 0.37 (0.32-0.42) | 0.34 (0.23-0.43) | 0.11 |
| RBP4 (mg/dL) | 3.5 (2.7-4.2) | 3.3 (2.8-4.0) | 3.5 (2.5-4.4) | 0.84 |
| Vitamin A/RBP4 ratio** | 0.73 (0.67-0.81) | 0.79 (0.72-0.85) | 0.70 (0.62-0.76) | < 0.001 |
| Ca (mg/dL) | 9.6 (9.3-9.8) | 9.5 (9.3-9.8) | 9.6 (9.2-9.8) | 0.35 |
| P (mg/dL) | 3.2 (2.9-3.6) | 3.3 (2.8-3.6) | 3.1 (2.9-3.6) | 0.64 |
| 25-OH Vitamin D (ng/dL) | 21.8 (15.6-30.9) | 24.1 (15.7-31.4) | 21.1 (15.0-29.4) | 0.14 |
| Vitamin E (mg/L) | 11.0 (9.6-13.0) | 11.3 (10-13.8) | 10.9 (8.9-12.8) | 0.11 |
| Vit. E/Cholesterol ratio (mg/g) | 6.4 (5.8-7.1) | 6.4 (5.9-7.0) | 6.3 (5.6-7.3) | 0.09 |
| Vit. E/total lipid ratio (mg/g) | 2.61 (2.34-2.87) | 2.72 (2.57-2.99 | 2.41 (2.19-2.73) | 0.029 |
| LBP (μg/dL) | 7.27 (5.72-8.57) | 6.73 (5.34-7.83) | 7.61 (6.43-10.03 | 0.018 |
| IL6** (pg/mL) | 3.51 (2.57-5.62) | 2.98 (1.80-4.01) | 4.72 (2.89-6.46) | 0.003 |
| HCV genotype (%)  1a  1b  2  3  4 | 11 (14.5)  54 (71.1)  1 (1.2)  5 (6.6)  5 (6.6) | 8 (23.5)  19 (55.9)  0  2 (5.9)  5 (14.7) | 3 (7.1)  35 (83.3)  1 (2.4)  3 (7.1)  0 |  |
| 3 vs. non-3 viral genotype | 5/71 | 2/32 | 3/39 | 0.82 |
| Viral load (low/high)*** | 14/62 | 8/26 | 6/36 | 0.38 |
| DBP phenotype Gc1s/Gc1s (yes/no) | 23/53 | 6/28 | 17/25 | 0.031 |
| *PNPLA3* rs738409 genotype  CC/CG/GG, n (%) | 40 (52.6)/28 (36.8)/8 (10.5) | 19 (55.9)/12 (35.3)/3 (8.8) | 21 (50.0)/16 (38.1)/5 (11.9) | 0.61 |
| *PNPLA3* rs738409 G allele carrier,  n (%) | 36 (47.4) | 15 (44.1) | 21 (50.0) | 0.61 |
| *IL28B* rs12979860 genotype  CC/CT/TT, n (%) | 17 (22.4)/43 (56.6)/16 (21.1) | 7 (20.6)/24 (70.6)/3 (8.8) | 10 (23.8)/19 (45.2)/13 (31.0) | 0.22 |

*Continuous variables are shown as median (interquartilic range). ** Values **not normally** distributed. *** See text. BMI: Body mass index. RBP4: Retinol-binding protein 4. LBP: Lipopolysaccharide-binding protein. IL6: Interleukin-6. DBP: Vitamin D binding protein. *PNPLA3*: Patatin-like phospholipase A3 gene. *IL28B*: Interleukin 28B gene.
